# Biomechanical analysis analyzing association between bone mineral density and lag screw migration

**DOI:** 10.1038/s41598-023-27860-5

**Published:** 2023-01-13

**Authors:** Hyeonjoon Lee, Soo Ah Kim, Sungmin Jo, Suenghwan Jo

**Affiliations:** 1 Department of Orthopedic Surgery, Chosun University Hospital, Gwangju, South Korea; 2 School of Medicine, Chosun University, 365 Pilmundae-Ro, Dong-Gu, Gwangju, 61453 South Korea; 3 Osteoporosis Study Group, Chosun University, Gwangju, South Korea; 4 Department of Obstetrics and Gynecology, Chosun University, Gwangju, South Korea

**Keywords:** Trauma, Medical research

## Abstract

A proximal femoral nail using a helical blade (HB) is commonly utilized to treat proximal femoral fracture but cut through failure of the lag screws is one of the devastating complications following the surgery. While controversial, one of the potential risk factors for cut through failure is poor bone strength which can be predicted by measuring bone mineral density (BMD). In this study, we performed a biomechanical test on the fractured femoral head to validate whether the indirectly measured BMD from the contralateral hip or that measured directly from the retrieved femoral head can elucidate the structural strength of the fractured femoral head and thereby can be used to predict migration of lag screws. Our result showed that directly measured BMD has a significant correlation with the HB migration on the osteoporotic femoral head. However, while the BMDs measured from the contralateral femoral neck or total hip is the most widely used parameter to predict the bone strength of the fractured femur, this may have limited usability to predict HB migration.

## Introduction

Proximal femoral fracture is one of the most common and devastating consequences of osteoporosis^1,2^. It is estimated that 1.7 million osteoporotic proximal femoral fractures occur worldwide annually, with mortality rates ranging from 22 to 29%^3,4^. For their treatment, proximal femoral nails are commonly accepted as an implant of choice. While surgery is mostly successful, failure of lag screws is one of the most severe complications. The typical failure mechanism occurs when the lag screw penetrates or cuts the femoral head; this is termed as “cut through” or “cut out” depending on the type of the lag screw used and on the direction of how the penetration occurs^5–8^.

A few hypotheses have been suggested to explain why such a phenomenon occurs and to prevent such failure. While controversial, one of the potential risk factors is poor bone strength^9,10^. As the osteoporotic bone may lack mechanical strength, the bone structure of the femoral head may not provide sufficient support, leading to the migration of the lag screw, which may result in the penetration of the femoral head. As bone strength is difficult to measure in vivo, bone mineral density (BMD) is commonly used to quantify and predict the strength of the osteoporotic bone^11^. In cases of a fractured proximal femur, measurements are provided indirectly from the contralateral hip or the spine. However, there is a paucity of literature on whether indirectly measured BMD from these regions can accurately reflect the mechanical strength of the fractured femoral head. Similarly, it is unclear whether we can predict the excessive migration of helical blade (HB) type of lag screws from this information.

Therefore, in this study, we performed a biomechanical test on the osteoporotic femoral head to validate whether the indirectly measured BMD from the contralateral or that directly measured from the retrieved femoral head can elucidate the structural strength of the fractured femoral head and thereby can be used to predict migration of lag screws. More specifically, the current study aims to assess (1) whether the BMD of the fractured femur correlates with the resistance to the HB migration on the fractured femoral head, and (2) whether the BMD of the contralateral hip can be used to predict excessive HB migration.

## Methods

The experimental protocol of this study was approved by our institutional review board before the experiment was conducted (CHOSUN #2020-03-010-0001). All methods were carried out by relevant guidelines and regulations and informed consent was obtained from patients or their legal guardians before the femoral head was retrieved. The femoral head was retrieved from female patients who underwent hip arthroplasty owing to femoral neck fracture between March 2018 and June 2021. The patients were given the option to undergo either arthroplasty or primary fixation and the decision was made by the patients and their legal guardians after a detailed description of both surgery's advantages and disadvantages^12^. The specimens from the patients who agreed to donate the retrieved femoral head for the purpose of this study were selected for analysis. The femoral heads were excluded if acquired from patients (1) who had underlying pathologic conditions that may influence bone quality other than osteoporosis, (2) who had undertaken medication that may potentially influence the quality of the bone, (3) whose BMD was not obtained from the contralateral hip owing to the remaining implants from previous surgery, and 4) with a history of osteoporotic fractures elsewhere.

Thirty-two femoral heads that fulfilled our inclusion and exclusion criteria were retrieved, which constituted the basis of our study. Nineteen femoral heads were from the right-side hip. The demographic data of the donors are listed in Table 1.

**Table 1:** Demographic data of the donor patients.

	Mean ± standard deviation	Minimum/maximum
Age (years)	78.20 ± 10.21	50/94
Time from fracture to retrieval (days)	4.71 ± 3.17	1/7
Body mass index (kg/m^2^)	21.76 ± 3.21	16.47/28.47
Femoral head diameter (mm)	47.45 ± 3.49	42/56

### BMD measurement of the non-fractured hip

**Figure 1:**
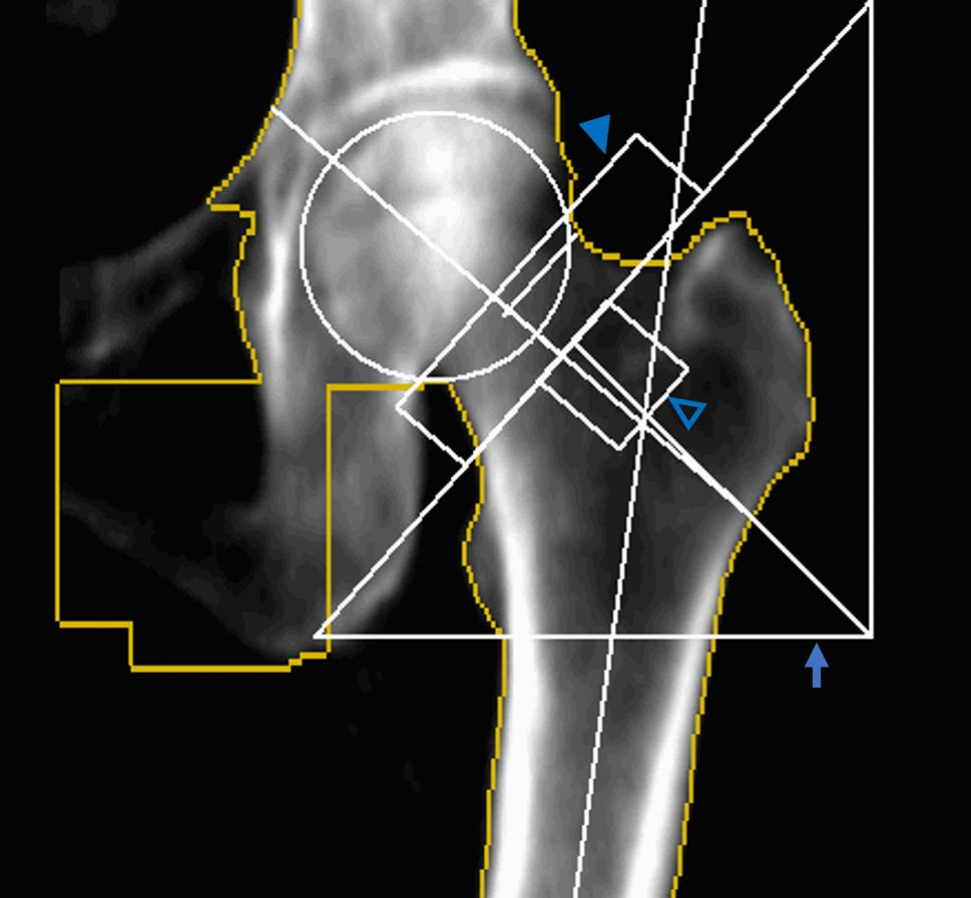
BMD was measured with DXA in the contralateral hip. ROI for the femoral neck (solid arrowhead), ward’s triangle (hollow arrowhead), and trochanter (arrow) are shown. ROI for the total hip is sum of the three ROIs.

The BMD of the donor patients was measured from the non-fractured hip using dual-energy X-ray absorptiometry (DXA; Prodigy Advance, GE Healthcare, USA) at the time of admission. The patient was positioned with a non-fractured hip in 15 degrees of internal rotation which provides the greatest area for measurement^13^. While BMDs from several regions can be measured, the BMDs of the total hip region and the neck region were used for analysis as these are the measurements commonly used clinically^14^. The BMDs were measured and collected using the Encore program (GE Lunar Prodigy, USA), with the region of interest (ROI) set automatically by the software built in the DXA scanner and adjusted by the radiotechnologist when necessary (Fig. 1).

All measurements were performed by two radiotechnologists and the mean values were used for analysis. Quality control was performed for both technologists and densitometry devices according to the protocol recommended by the International Society for Clinical Densitometry (ISCD)^15^. Precision assessment was also performed before the experiment to measure the least significant difference (LSC), which was 4.2% in the total hip and 5.1% in the femoral neck. The measured LSC in our institution is within the recommendation of the ISCD.

### Retrieval of the femoral head

At the time of hip arthroplasty, the femoral head was retrieved with caution to minimize iatrogenic damage. If the ligamentum teres were intact, it was resected from fovea capitis, an oval-shaped dimple in the superior-medial portion of the femoral head, with a scalpel to prevent avulsion of the femoral head during the dislocation process. The retrieved femoral head was washed with saline and dried at room temperature for 1 h. This was then fresh frozen at − 20 °C for later experiments.

### BMD measurement of the fractured hip

Direct BMD of the retrieved femoral head was measured using the Quantum GX micro-computed tomography (micro-CT) imaging system (PerkinElmer, Hopkinton, MA, USA), located at Korea Basic Science Institute (Gwangju, Korea). The decision was made to use micro-CT over DXA as there is currently no standardized way of measuring BMD with DXA when the femoral head is not in its anatomical position. Also, micro-CT has been validated for its high accuracy in measuring BMD^16^. The fresh frozen femoral head was thawed at room temperature for 24 h before the micro-CT scan. All measurement was made within 10 days of the retrieval.

**Figure 2:**
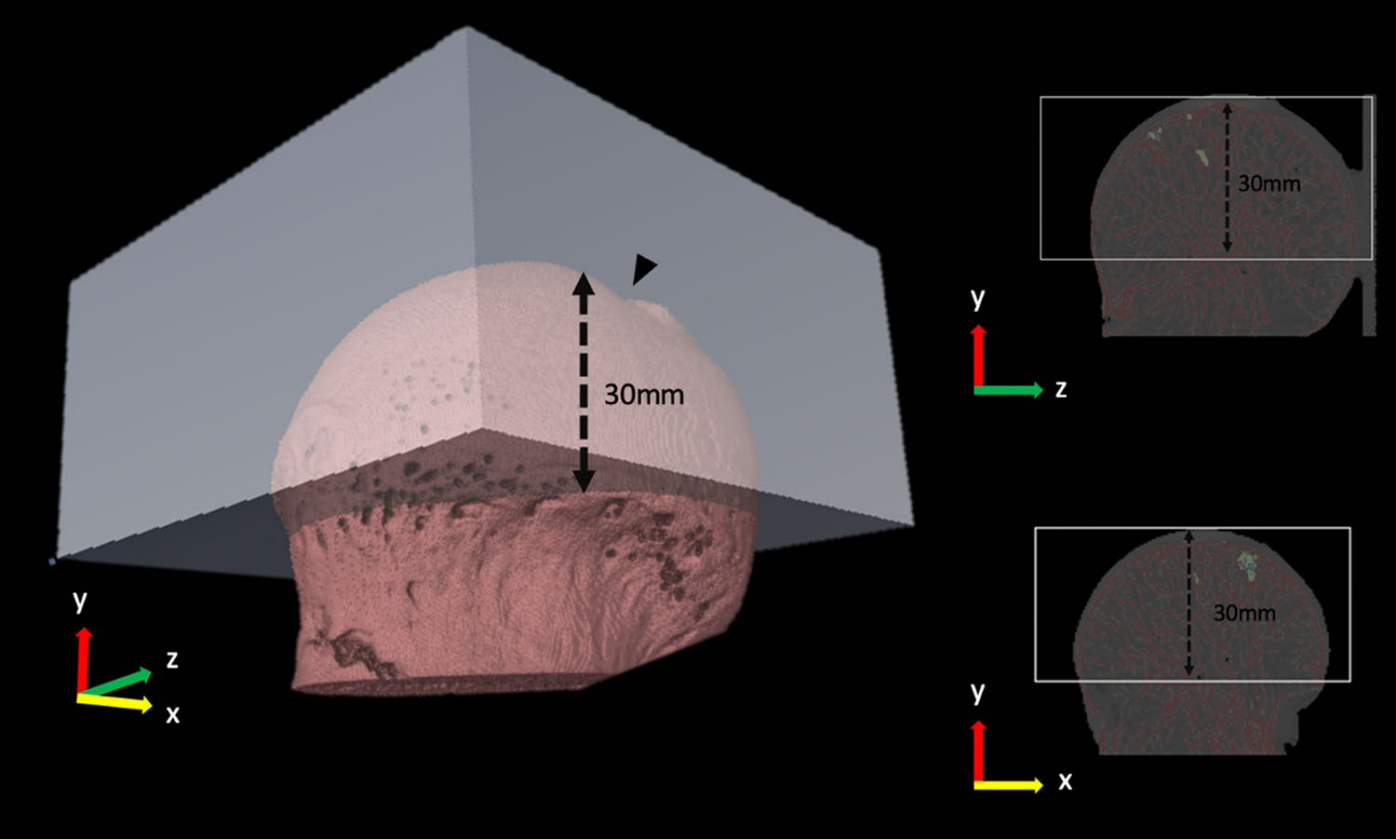
ROI of the femoral head when BMD was directly measured with micro-CT. A spherical cap with 30 mm height was used as this was the volume typically preserved after the retrieval process. Arrowhead indicates the location of fovea capitis which was used as a landmark.

For the scanning process, the X-ray source was set to levels of 90 kV and 88 μA with a field of view of 72 mm and a slice thickness of 0.144 mm. The scanning time was 4 min in a 360° rotation. Using AccuCT™ analysis software (PerkinElmer, USA), all raw CT values were converted to Hounsfield Units (HU). The intensity of water was defined as 0 HU and that of dry air as − 1000 HU. The calibration of the AccuCT™ analysis software was performed with the use of hydroxyapatite (HA) phantom (QRM-Micro-CT-HA, Quality Assurance in Radiology and Medicine GmbH, Germany). The spherical cap region of 30 mm from the fovea capitis was selected as an ROI of the retrieved femoral head (Fig. 2). The ROI was selected based on the maximum volume of the femoral head preserved after the retrieval procedure.

### Femoral head preparation

Immediately following the BMD measurement, the femoral head was fixed to the custom-made jig, which was used as a guide for excessive bone resection and stabilization during the mechanical test. The jig was manufactured with stainless steel and includes a spherical cap engraving of 20 mm height to adopt the femoral head. The 20 mm height was determined as the tip of the HB is typically placed 10 mm from the outer surface of the femoral head during the surgery and we added an additional 10 mm to accommodate the potential surgical error. The jig also includes two holes to insert 2.8-mm K-wires, so that the femoral head is firmly fixed within the engraving. An additional hole was developed at the inferior region of the engraved sphere to enable penetration of the lag screw during the biomechanical test. Four jigs with the same design were manufactured with a diameter of the engraved hemisphere in 4 mm increments from 40 to 56 mm so that different sizes of the femoral head could be adopted (see Supplementary Fig. 1).

**Figure 3:**
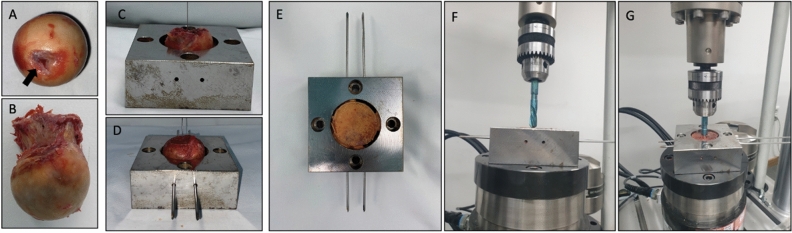
Retrieved femoral head (**A**, **B**) is placed in the jig (**C**) and stabilized with multiple k-wires (**D**). The protruding part of the femoral head is resected (**E**) leaving femoral head with 20 mm height for the mechanical test. After fixating jig to the universal test machine, the lag screw is adjusted so that the tip is in contact with the resected surface of femoral head (**F**). The lag screw is advanced for 25 mm (**G**) which results in 5 mm cut through on the femoral head. Arrow indicates fovea capitis.

Based on the trajectory of a typical lag screw, the femoral head was fixated in the position where the region 5 mm above the fovea capitis is placed at the inferior most part of the sphere cap engraving. The position was confirmed through the hole at the bottom of the jig with fovea capitis used as a landmark. After positioning of the femoral head, the protruded bone out of the jig was resected, leaving a sphere cap of a 20-mm height for biomechanical testing (Fig. 3A–E).

### Measuring resistance to helical blade migration

The mechanical test was performed using a servo-hydraulic universal test machine (MTS Bionix Landmark 370, MTS System Corporation, USA). A helical blade-type lag screw from a commercially available proximal femoral nail system (PFNA-II blade, Depuy Synthes, Switzerland) was utilized to test the resistance properties to the helical blade migration. The specifications from the provider indicated that the PFNA-II helical blade has a diameter of 12.2 mm. We used lag screws of 85 mm in length for the experiment. For the setup, lateral locking of the PFNA-II helical blade was released, so that the blade portion of the PFNA-II lag screw can be freely rotated. This was determined to reproduce the failure mechanism of the lag screw where the femoral head is typically rotated along the lag screw^17,18^.

Initially, the position of the lag screw was manually adjusted so that the tip is in contact with the resected surface of the femoral head. This was then advanced at 15 mm/min until the lag screw penetrated the femoral head and advanced for an additional 5 mm to replicate the cut through situation (Fig. 3F, G). The load–displacement curve was acquired during the 25-mm advancement of the lag screw.

The resistance properties were defined as (1) peak resistance and (2) total resistance during the 25-mm advancement. The peak resistance was defined as the maximum load measured in the load–displacement curve, while the total resistance was defined as the area under the load–displacement curve during the 25-mm advancement of the lag.

### Statistical methods

The sample size was estimated a priori using G*power software (version 3.1.9.3, Heinrich-Heine-Universität Düsseldorf, Düsseldorf, Germany)^19^. With a correlation effect size of 0.5, an alpha error of 0.05, and a beta error of 0.2 to ensure power of 80%, the estimation indicates that it will be necessary to include at least 29 cases for the purpose of the current study. The measured results were expressed as means and standard deviations. The correlations between the following variables were assessed: (1) BMD of the contralateral hip and that of the fractured femoral head, (2) BMD of the fractured femoral head and resistance properties of the fractured femoral head, and (3) BMD of the contralateral hip and resistance properties of the fractured femoral head. The normality of the distribution of the data was assessed using the Kolmogorov–Smirnov test^20^. Correlation analysis was performed using Pearson correlation or Spearman rank correlation test according to the normality of the distribution of each variable. In addition, linear regression analysis was performed to confirm the change in mechanical resistance properties according to the BMD of the contralateral hip and the fractured femoral head.

Statistical analysis was performed using the SPSS software version 27 (SPSS Inc., IL, USA). All P-values were two-sided, and *P* values of < 0.05 were considered significant.

## Results

The mean interval from the time of the fracture to BMD measurement using DXA was 1.9 ± 1.2 days. The mean time from the retrieval of the fractured femoral head to micro-CT measurement and the mechanical test was 7.6 ± 3.3 days.

The mean BMD of the contralateral hip measured on DXA was 0.61 ± 0.15 g/cm^2^ in the femoral neck and 0.65 ± 0.16 g/cm^2^ in the total hip, which corresponds to a T-score of − 2.74 ± 1.21 and − 3.11 ± 1.34, respectively. The BMD of the fractured femoral head measured on micro-CT was 467.8 ± 69.3 mg HA/cm^3^. The measured BMDs and the correlation between the fractured and non-fractured sides are summarized in Tables 2 and 3.

**Table 2:** Bone mineral density of the fractured femoral head as measured by micro-CT and of the non-fractured contralateral hip as measured by dual-energy X-ray absorptiometry.

	Mean ± SD	Minimum	Maximum
Non-fracture hip (g/cm^2^)			
Total hip	0.65 ± 0.16	0.42	0.91
Femoral neck	0.61 ± 0.15	0.41	0.87
Fractured hip (g HA/cm^3^)			
Femoral head	0.4678 ± 0.0693	0.2734	0.6187

*SD* standard deviations.

**Table 3:** Correlation between the BMDs of the contralateral hip and that of the fracture femoral head.

		Correlation coefficient (*r*)	*p*
BMD neck(g/cm^2^)	Micro-CT(g HA/cm^3^)	0.330	0.530
BMD total hip(g/cm^2^)		0.286	0.096

The load–displacement curve during lag screw advancement through the femoral head showed an initial stiff increase followed by a gradual decrease (see Supplementary Fig. 2); however, there was wide variability among the specimens in terms of the peak and the total resistance. The resistance properties as measured by the peak resistance and the total resistance are listed in Table 4.

**Table 4:** Resistance properties of the fractured femoral head measured by mechanical testing.

	Mean ± SD	Minimum	Maximum
Peak resistance (kN)	1.20 ± 0.63	0.46	3.67
Total resistance (kN mm)	18.47 ± 9.24	7.09	52.34

*SD* standard deviations.

**Figure 4:**
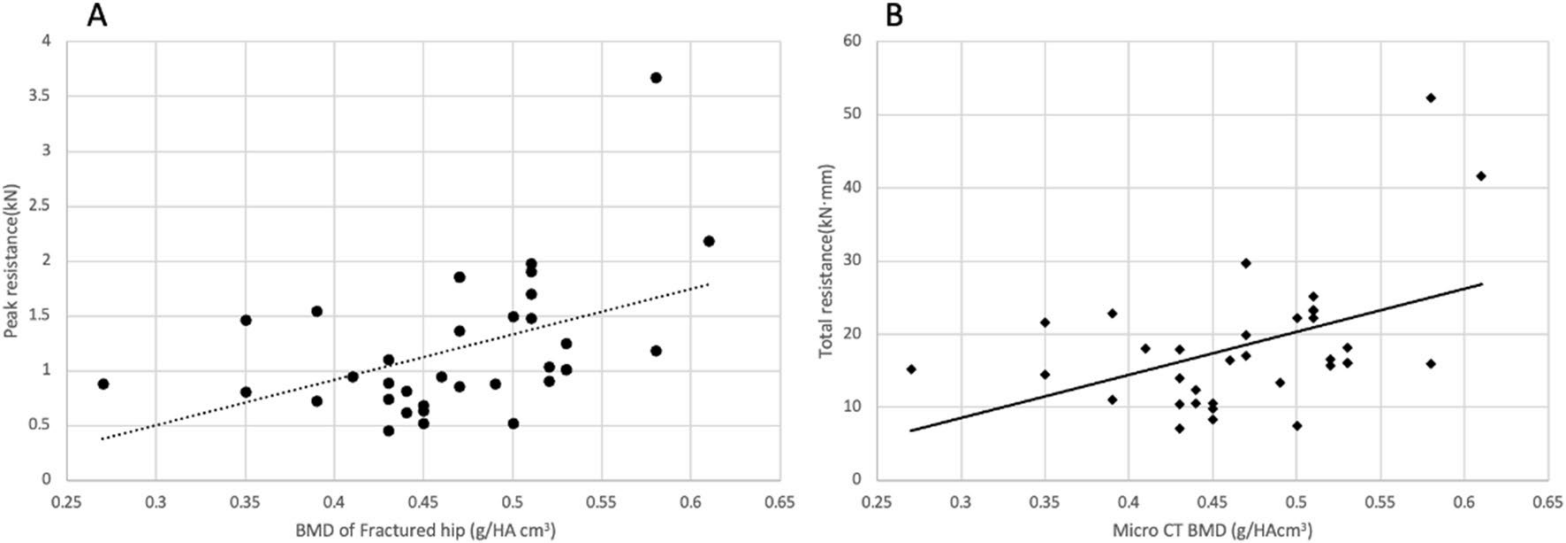
Scatter plot of the BMD of the fractured femoral hip and the resistance properties. (**A**) Correlation between the BMD of fractured femoral head and the peak resistance. (**B**) Correlation between the BMD of fractured femoral head and the total resistance.

When correlation was analyzed between measured BMDs and the resistance properties of HB on the femoral head, there was a significant positive correlation between the BMD and the resistance properties of the fractured femoral head (Peak resistance; *r* = 0.479, *p* = 0.004, Total resistance; *r* = 0.395, *p* = 0.019) (Fig. 4). However, no significant correlation was found between BMDs of the contralateral hip and resistance properties of the fractured femoral head. The correlation and linear regression analysis between the BMDs of the fractured and non-fractured sides and the resistance properties are summarized in Table 5.

**Table 5:** Correlation and linear regression analysis between the bone mineral densities and resistance properties.

Variable	Correlation analysis		Linear regression analysis	
	*r* Value	*p* Value	β Value	*p* Value
Peak resistance (kN)				
BMD neck (g/cm^2^)	0.192	0.269	0.441	0.558
BMD total (g/cm^2^)	0.202	0.245	0.425	0.554
BMD micro-CT (g HA/cm^3^)	**0.479**	0.004*	**4.134**	0.006*
Total resistance (kN mm)				
BMD neck (g/cm^2^)	0.168	0.335	7.469	0.497
BMD total (g/cm^2^)	0.162	0.353	4.560	0.665
BMD micro-CT (g HA/cm^3^)	**0.395**	0.019*	**58.970**	0.008*

*r*, correlation coefficient; *p,* significance; β, standardized coefficient.

Significant values are in bold.

## Discussion

The results of this study show that the BMD measured directly from the fractured femoral head may reflect resistance to the HB migration but that measured from the non-fractured hip does not correlate with the resistance to the HB migration on the fractured femoral head indicating that this may not be used to predict the excessive migration of the HB lag screw.

A proximal femoral nail with a sliding hip screw is commonly used to stabilize proximal femoral fractures with satisfactory outcomes but the failure of lag screws remains to be fatal complications^8,21^. For minimizing the risk of developing lag screw failure, a helical blade-design lag screw was developed to provide better rotational and varus stability by compacting the trabecular bone around the flanges of the blade^22^. However, a problem of medial migration remained which has been reported to be observed in 0.7–6.3% of patients undergoing surgery with this type of lag screw^6,23^. Several factors have been suggested to be a potential reason for this phenomenon, which include inappropriate tip–apex distance, unstable fracture pattern, and the z-effect phenomena^24,25^. One additional potential risk factor would be deficient bone strength of the femoral head^9^. Theoretically, the trabecular bone surrounding the helical blade may function to resist further advancement of the lag screw. Therefore, it can be hypothesized that in the femoral head with weak bone strength, resistance may not be sufficient to prevent medial migration^26,27^.

One of the most widely used methods to predict bone strength is measuring the BMD using DXA. However, the BMD largely represents the quantity of the trabecular bone. While bone mass is one of the important factors contributing to bone strength, the mechanical properties of the bone are also configured by other factors, such as architecture geometry, cortical porosity, and tissue mineralization density^28^. Therefore, Ammann et al. reported that DXA-measured BMD may predict only 60–70% of the variation in bone strength according to established studies that validated the correlation between the BMD and bone strength^29,30^.

We are aware of only two studies that investigated the relationship between the BMD and the mechanical properties of the femoral head. Haba et al.^31^ investigated the correlation between the mechanical properties and BMD of 22 femoral heads. The study performed uniaxial compression tests on the cylindrical bone samples which were retrieved from the osteoarthritic femoral head. They reported that there was a weak but significant correlation between the BMD and the mechanical properties of the trabecular bone. Interestingly, in the subsequent study by the same authors, only the structural modulus had a significant correlation with the BMD and not the ultimate compression strength^32^. Because of different test protocols, we cannot make a head-to-head comparison with these studies, but our findings are somewhat similar in that we found a significant correlation between the BMD and the mechanical properties of the femoral head as measured by the ultimate and total resistance of the HB-type lag screw. On the other hand, our study also showed that the BMDs of the contralateral hip do not reflect the resistance properties of the fractured femoral head as measured by the HB migration. This is likely because the BMD of the fractured head does not correlate with the BMDs of the contralateral hip which is another finding of our study.

A number of studies have reported a correlation of the BMD from the bilateral hip with conflicting results. Banse et al.^33^ compared the mechanical properties of 10 paired left–right proximal femurs and reported that no significant difference was found when both sides were compared. Conversely, a larger study by Afzelius et al.^34^ measured the BMD of the bilateral hip in 133 participants and reported that while there was no difference when the BMD of the femoral neck was compared, the total hip BMD was lower in the dominant leg. Another study by Li et al.^35^ compared the BMD of the non-fractured hip side and fractured hip side using quantitative CT. They reported that the volumetric BMD of the non-fractured side was higher than that of the fractured side and that the difference was significant when the BMD was measured through the center of the femoral neck. In our study, the BMD of the non-fractured side was measured using DXA with the ROI in the femoral neck and total hip while that of the fractured side was measured at the femoral head using micro-CT. It should be noted that the typical measurement of the total hip BMD does not include the femoral head, as this image overlaps with the posterior acetabular wall, a bony structure located in the posterior part of the hip socket. Therefore, our result arises from the comparison among different parts of the proximal femur which may be the potential reason why no correlation was found. Nonetheless, we believe this inconsistency of BMD in the bilateral hip may be the explanation for why BMD of the contralateral hip does not correlate with the resistance properties measured in the fractured femoral head.

We acknowledge that there are a number of limitations to our study. First, the study is largely limited by the number and characteristics of the specimens. The retrieved femoral heads were mostly from elderly patients with an osteoporotic femoral neck fracture, and it is unclear whether the current conclusion is applicable to younger patients with stronger bone. Therefore, data from different age groups and a wide range of BMD would provide us with a better understanding of the relationship between the BMD and the mechanical strength of the femoral head. Second, our study tested the axial compression load of the HB on the femoral head but the direction of the lag screw in the study does not correspond to the physiologic load applied in vivo. Owing to the anatomical axis of the lower leg, the load against the femoral head should be applied to the superior-medial direction toward the acetabulum but we were unable to reproduce this in our study^36^. Similarly, the factors that may influence the outcome in real patients, such as body mass index or walking habits, are not considered in our study design. Therefore, the application of our results to actual practice should be made with caution. Another limitation is the potential effect of freezing and thawing on the specimens. After retrieval of the femoral head, we froze the samples at − 20 °C until we had access to micro-CT and the MTS. Numerous studies have shown that the freeze and thaw process has a minimal effect on the cortical bone; however, there is limited evidence of its effect on the trabecular bone^37,38^. We think the immediate test on the femoral head following retrieval may have provided results that may more accurately imitate the condition of the femoral head in the body.

Nevertheless, this is the first study to validate the correlation between BMDs and the resistance to HB migration on the femoral head.

## Conclusions

Our study indicates that directly measured BMD has a significant correlation with HB migration on the osteoporotic femoral head. However, while the BMD measured from the non-fractured contralateral femoral neck or total hip using DXA is the most widely used parameter to predict the bone strength of the fractured femur, our findings suggest that this may have limited usability to predict helical blade migration.

## Supplementary Information

Supplementary Information 1.

Supplementary Information 2.

## Data Availability

The datasets generated during and/or analyzed during the study are available from the corresponding author on request.

## Abbreviations

BMD: Bone mineral density
HB: Helical blade
DXA: Dual-energy X-ray absorptiometry
ROI: Region of interest
ISCD: International Society for Clinical Densitometry
LSC: Least significant difference
CT: Computed tomography
