# Supplementary material for: Biomechanical analysis analyzing association between bone mineral density and lag screw migration
Source: Sci Rep. 2023 Jan 13;13:747. doi: 10.1038/s41598-023-27860-5 (PMC9839704; doi:10.1038/s41598-023-27860-5)

Supplementary figure 1. A schematic design of jig as seen from above (A) and from front (B). An actual jig was manufactured with stainless steel (B). Height of the engraving (h) was set to 20mm, and the 4 jigs with different diameter (d) was manufactured to accommodate femoral head with different size. Arrow heads indcate holes for k-wire.


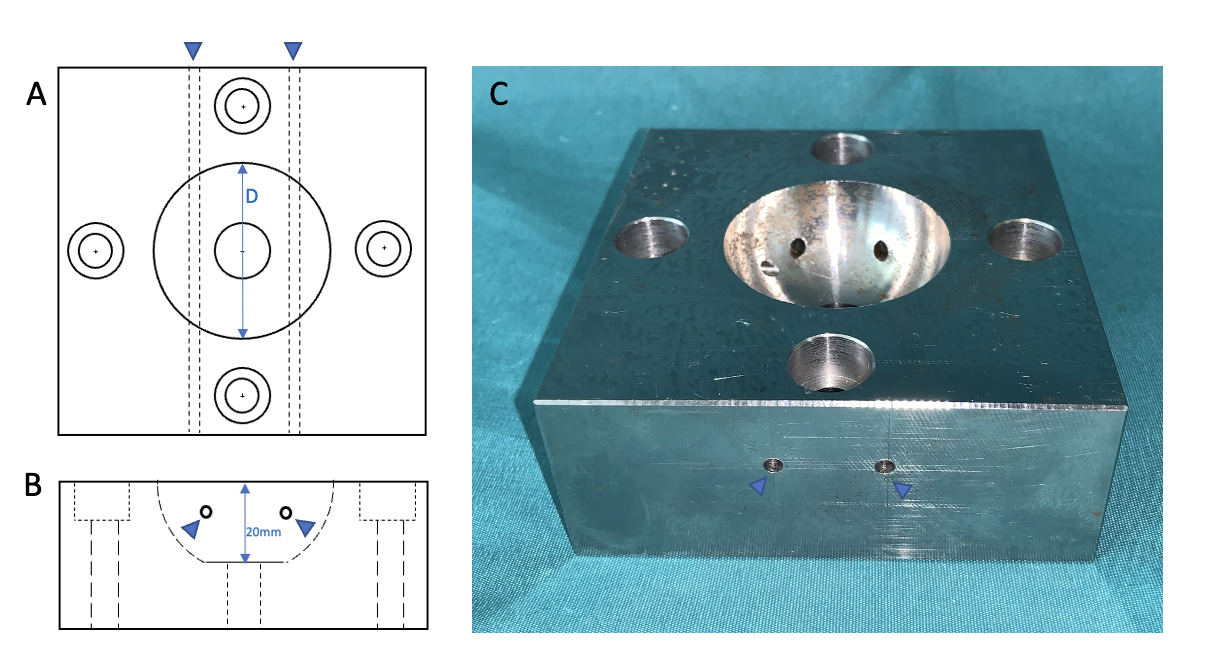

Supplement: Supplementary file 1 — Supplementary Information 1. [file 41598_2023_27860_MOESM1_ESM.docx]
