# Supplementary material for: Biomechanical analysis analyzing association between bone mineral density and lag screw migration
Source: Sci Rep. 2023 Jan 13;13:747. doi: 10.1038/s41598-023-27860-5 (PMC9839704; doi:10.1038/s41598-023-27860-5)

Supplementary figure 2. A typical load-displacement curve during 25mm lag screw advancement through the femoral head shows an initial stiff increase followed by a gradual decrease. The peak resistance was defined as the maximum load measured in the load-displacement curve (arrow), while the total resistance was defined as the area under the load-displacement curve.


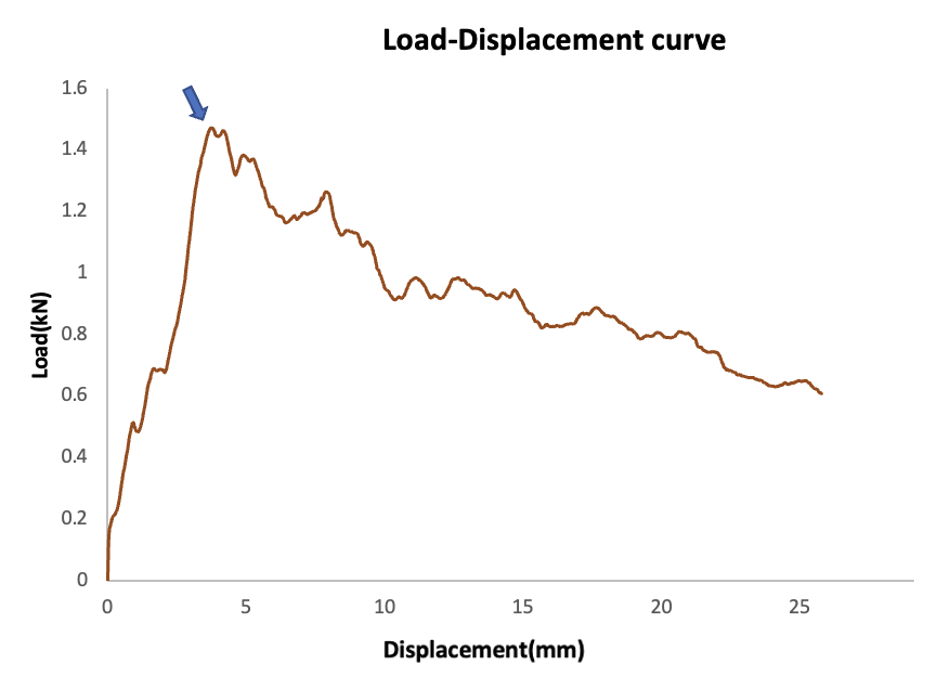

Supplement: Supplementary file 2 — Supplementary Information 2. [file 41598_2023_27860_MOESM2_ESM.docx]
